# External validation of the PROLOGUE score to predict neurological outcome in adult patients after cardiac arrest: a prospective cohort study

**DOI:** 10.1186/s13049-023-01081-1

**Published:** 2023-04-04

**Authors:** René Blatter, Bulus Gökduman, Simon A. Amacher, Christoph Becker, Katharina Beck, Sebastian Gross, Kai Tisljar, Raoul Sutter, Hans Pargger, Stephan Marsch, Sabina Hunziker

**Affiliations:** 1 Medical Communication and Psychosomatic Medicine, University Hospital Basel, Klingelbergstrasse 23, 4031 Basel, Switzerland; 2 Intensive Care Unit, University Hospital Basel, Basel, Switzerland; 3 Department of Emergency Medicine, University Hospital Basel, Basel, Switzerland; 4 Medical Faculty, University of Basel, Basel, Switzerland

**Keywords:** Cardiac arrest, Cardiopulmonary resuscitation, Neurological prognostication, Prognostic model, PROLOGUE, OHCA, CAHP

## Abstract

**Background:**

The PROLOGUE score (PROgnostication using LOGistic regression model for Unselected adult cardiac arrest patients in the Early stages) is a novel prognostic model for the prediction of neurological outcome after cardiac arrest, which showed exceptional performance in the internal validation. The aim of this study is to validate the PROLOGUE score in an independent cohort of unselected adult cardiac arrest patients and to compare it to the thoroughly validated Out-of-Hospital Cardiac Arrest (OHCA) and Cardiac Arrest Hospital Prognosis (CAHP) scores.

**Methods:**

This study included consecutive adult cardiac arrest patients admitted to the intensive care unit (ICU) of a Swiss tertiary teaching hospital between October 2012 and July 2022. The primary endpoint was poor neurological outcome at hospital discharge, defined as a Cerebral Performance Category (CPC) score of 3 to 5 including death.

**Results:**

Of 687 patients included in the analysis, 321 (46.7%) survived to hospital discharge with good neurological outcome, 68 (9.9%) survived with poor neurological outcome and 298 (43.4%) died. The PROLOGUE score showed an area under the receiver operating characteristic curve (AUROC) of 0.83 (95% CI 0.80 to 0.86) and good calibration for the prediction of the primary outcome. The OHCA and CAHP score showed similar performance (AUROC 0.83 and 0.84 respectively), the differences between the three scores were not significant (*p* = 0.495). In a subgroup analysis, the PROLOGUE score performed equally in out-of-hospital and in-hospital cardiac arrest patients whereas the OHCA and CAHP score performed significantly better in OHCA patients.

**Conclusion:**

The PROLOGUE score showed good prognostic accuracy for the early prediction of neurological outcome in adult cardiac arrest survivors in our cohort and might support early goals-of-care discussions in the ICU.

*Trial registration* Not applicable.

**Supplementary Information:**

The online version contains supplementary material available at 10.1186/s13049-023-01081-1.

## Background

Potentially reversible cardiac arrest is a major public health issue faced by patients and health systems worldwide [1–3]. Progress in post-resuscitation care has improved survival significantly [4], but neurological sequelae due to hypoxic-ischaemic brain injury remain a concern among patients with return of spontaneous circulation (ROSC) after cardiac arrest [5, 6]. Early prognostication of neurological outcome in cardiac arrest survivors however remains difficult [7]. Current guidelines recommend to delay neurological prognostication in comatose cardiac arrest patients until 72 h after ROSC [8]. In order to provide additional guidance for discussions about goals of care and the extent of therapeutic effort, several scoring models have been developed, which use different clinical and laboratory values to calculate the probability of poor neurological outcome [9, 10]. This probability might be integrated into an overall clinical judgment using professional experience, clinical and neurological assessment.

Two of the most promising and thoroughly validated scoring models for the prognostication of neurological outcome after cardiac arrest are the Out-of-Hospital Cardiac Arrest (OHCA) and the Cardiac Arrest Hospital Prognosis (CAHP) scores [9, 11–13]. Both of these scores have shown good prognostic accuracy in numerous previous validations [13–30]. A drawback of both models is the fact that they require no-flow time, which is often inaccurate or unknown, especially if the cardiac arrest was unwitnessed. A South-Korean research group recently developed the PROLOGUE (PROgnostication using LOGistic regression model for Unselected adult cardiac arrest patients in the Early stages) score to address this issue [14]. The PROLOGUE score includes resuscitation, clinical and laboratory parameters and omits the no-flow duration as a predictor variable. Box 1 gives an overview of the parameters included in the OHCA-, CAHP-, and PROLOGUE score. In the internal validation and one external validation, both conducted in South Korea, the PROLOGUE score showed excellent prognostic performance with areas under the receiver operating characteristic curve (AUROC) of 0.94 and 0.92 respectively [14, 30]. Furthermore, the probability of poor neurological outcome can easily be calculated at the bedside using a nomogram provided in the original publication. The PROLOGUE score therefore seems like a promising new scoring model to assist with early prognostication after cardiac arrest. However, a recent Austrian validation study including 1051 adult cardiac arrest patients failed to reproduce the excellent performance of the score in the Korean publications [31]. This highlights the need for further external validation in different settings and countries. Therefore, the aim of this study is to provide an independent validation of the PROLOGUE score in a large European cohort of unselected adult cardiac arrest survivors and to compare it to the thoroughly validated OHCA and CAHP scores.

**Box 1:** Description of included scores

Score	Outcome predicted	Variables	Score calculation
OHCA	Poor neurological outcome (CPC 3–5)	Initial rhythm: VF or VT [yes/no]	−13 if no
		No-flow interval [min]	+ 6 × ln(no-flow interval)
		Low-flow interval [min]	+ 9 × ln(low-flow interval)
		Serum creatinine [µmol/L]	−1434/(serum creatinine)
		Arterial lactate [mmol/L]	+ 10 × ln(arterial lactate)
CAHP	Poor neurological outcome (CPC 3–5)	Age [years]	Points attributed by nomogram for all variables
		Arrest setting [home/public]	
		Shockable rhythm [yes/no]	
		No-flow interval [min]	
		Low-flow interval [min]	
		pH at admission	
		Dosage of epinephrine administered [0, 1–2 or ≥ 3 mg]	
PROLOGUE	Poor neurological outcome (CPC 3–5)	Unwitnessed collapse	Points attributed by nomogram for all variables
		Potassium ≥ 4.4 mEq/L	
		Lactate ≥ 8 mmol/l	
		Adrenaline dose ≥ 2 mg	
		Low-flow duration ≥ 18 min	
		Haemoglobin < 13.2 g/dl	
		Creatinine ≥ 1.21 mg/dl	
		Phosphate ≥ 5.8 mg/dl	
		Non-shockable rhythm	
		Absent pupillary light reflex	
		Age ≥ 59 years	
		Glasgow Coma Scale motor score < 2	

*CAHP* Cardiac arrest hospital prognosis score, *CPC* Cerebral performance category scale, *ln* logarithmus naturalis, *OHCA* out-of-hospital cardiac arrest score, *PROLOGUE* prognostication using logistic regression model for unselected adult cardiac arrest patients in the early stages, *VF* ventricular fibrillation, *VT* ventricular tachycardia

## Methods

### Study setting

We analysed prospectively collected data of adult cardiac arrest patients who were included in the COMMUNICATE/PROPHETIC cohort between October 2012 and July 2022 at the University Hospital Basel, a tertiary teaching hospital in Switzerland. The details of the study have been published previously [32]. Informed consent was obtained from the patients or from their relatives, depending on the patient's decision-making capacity. The study was approved by the Ethics Committee of North-western and Central Switzerland (www.eknz.ch) and followed the principles of the Declaration of Helsinki and its amendments. Analysis and reporting for this study were conducted in accordance with the Transparent Reporting of a Multivariable Prediction Model for Individual Prognosis or Diagnosis (TRIPOD) statement [33, 34].

### Participants

The COMMUNICATE/PROPHETIC registry included unselected cardiac arrest patients ≥ 18 years of age treated in the ICU of the University Hospital Basel. Eligible were all patients with ROSC after out-of-hospital (OHCA) or in-hospital (IHCA) cardiac arrest. Excluded were patients suffering a cardiac arrest while being monitored (e. g., ICU, intermediate care unit, operating theatre, cardiac catheterisation laboratory) and patients where informed consent was denied. The treatment of the patients was conducted according to the standardised local treatment protocol and followed the respective current guidelines of the European Resuscitation Council [8, 35, 36].

### Outcomes

The primary endpoint was neurological outcome at hospital discharge as measured by the Cerebral Performance Category (CPC) scale [37]. The CPC scale differentiates five levels of functional outcome: A score of 1 indicates good recovery with resumption of normal life, a score of 2 indicates moderate disability with independence concerning daily life, a score of 3 indicates severe disability with the need of daily support, a score of 4 indicates a persistent vegetative state and a score of 5 equals death or brain death [37]. A CPC score of 1 to 2 was defined as good, a score of 3 to 5 as poor neurological outcome. The secondary outcome was in-hospital mortality.

### Data collection

The following data were extracted for each patient from the electronic patient records: Sex category (as assigned at birth or as reported by the patients/relatives), age, pre-existing chronic diseases (coronary artery disease, heart failure, neurologic disease, diabetes, hypertension, chronic obstructive pulmonary disease, chronic kidney disease, liver cirrhosis and malignancy), resuscitation parameters (location of arrest, presence of a witness to the collapse, if bystander cardiopulmonary resuscitation [CPR] was performed, first monitored rhythm, dose of epinephrine [adrenaline] administered during CPR, no-flow duration, low-flow duration), cause of cardiac arrest, clinical and laboratory parameters at ICU admission (Glasgow Coma Scale [GCS] including the three sub-scores, presence of pupillary light reflex, c-reactive protein, blood glucose level, blood pH, lactate, phosphate, potassium, sodium, haemoglobin, creatinine), interventions performed during the ICU stay (mechanical ventilation, coronary angiography, administration of vasoactive agents and TTM), and CPC score at hospital discharge.

### Score risk categories

The OHCA and CAHP scores were categorised as described by previous publications: The OHCA score was divided into four categories (≤ 20; > 20–40; > 40–60; > 60 points) [18], the CAHP score into three categories (< 150; 150–200; > 200 points) [12]. For the PROLOGUE score no such categories have been suggested. Instead, we assessed prognostic accuracy at each decile of predicted risk in accordance with the original publication [14].

### Statistical analysis

Continuous variables were described using mean and standard deviation (SD) or median and interquartile range (IQR), categorical and binary variables were described by counts and proportions. Continuous variables were checked visually for normality of distribution using Q-Q-Plots. For comparison between groups Pearson’s χ^2^-Test (binary and categorical variables), ANOVA (continuous, normally distributed variables) and the Wilcoxon rank-sum test (continuous, skewed variables) were applied as appropriate. The PROLOGUE-, OHCA-, and CAHP-score values were calculated as indicated in the original publications. PROLOGUE-. OHCA-, and CAHP-scores’ prognostic performance was assessed using measures of discrimination and calibration. Discriminatory performance was analysed using the area under the receiver operating curve (AUROC). An AUROC of 0.7–0.8 was defined as acceptable, an AUROC of 0.8–0.9 as good and > 0.9 as excellent. Comparison of AUROC between PROLOGUE-, OHCA-, and CAHP-scores was conducted using the approach by DeLong et al.[38] Sensitivity, specificity, positive predictive value (PPV) and negative predictive value (NPV) were assessed for the cut-off values (i.e., categories) specified in the ‘Score Risk Categories’ section. Calibration was assessed graphically using a calibration plot depicting the event rates predicted by the respective score vs. the event rates observed in our cohort. Subgroup analysis comparing the PROLOGUE-, OHCA-, and CAHP-scores' performances in IHCA vs. OHCA patients as well as in female vs. male sex category was conducted. A two-sided *p*-value of < 0.05 was considered to represent statistical significance.

## Results

### Baseline characteristics

From 708 eligible patients with ROSC after cardiac arrest, 21 patients were excluded due to screening failure or missing informed consent. Six-hundred-eighty-seven patients were included in the final analysis. The baseline characteristics of our cohort are shown in Table 1 along with the baseline characteristics of the original PROLOGUE development cohort. Factors significantly associated with poor neurological outcome were higher age, male gender, chronic comorbidities (coronary artery disease, chronic obstructive pulmonary disease, diabetes, cancer, neurological disease), longer no-flow and low-flow durations, unwitnessed cardiac arrest, no bystander CPR, non-shockable initial rhythm, higher dose of adrenaline administered before ROSC, non-cardiac cause of cardiac arrest, as well as lower GCS score, non-reactive pupils, higher levels of C-reactive protein, creatinine, blood glucose, phosphate and lactate, lower pH and lower haemoglobin at ICU admission.

**Table 1:** Baseline characteristics of the study population and comparison with the development cohort

	PROLOGUE development cohort[14]	COMMUNICATE/PROPHETIC cohort			
	All	All	CPC 1–2	CPC 3–5	*p*-value
n	671	687	321	366	
*Sociodemographics*					
Age, years, median (IQR)	63 (52–74)	65.5 (55.8–75.8)	62.3 (53.3–72.9)	69.3 (57.9–78.2)	< 0.001
Male gender, n (%)	453 (67.5)	493 (71.8)	252 (78.5)	241 (65.8)	< 0.001
*Comorbidities, n (%)*					
Hypertension	270 (40.2)	355 (51.7)	167 (52.0)	188 (51.5)	0.89
Coronary artery disease	84 (12.5)	399 (58.2)	206 (64.2)	193 (52.9)	0.003
Congestive heart failure	32 (4.8)	96 (14.0)	38 (11.8)	58 (15.9)	0.12
COPD/pulmonary disease	49 (7.3)	76 (11.1)	18 (5.6)	58 (15.9)	< 0.001
Diabetes	183 (27.3)	149 (21.7)	58 (18.1)	91 (24.9)	0.03
Chronic kidney disease	66 (9.8)	93 (13.6)	38 (11.8)	55 (15.1)	0.22
End-stage liver disease	16 (2.4)	19 (2.8)	5 (1.6)	14 (3.8)	0.07
Malignant disease	62 (9.2)	75 (10.9)	22 (6.9)	53 (14.6)	0.001
Neurological disease	26 (3.9)	97 (14.1)	29 (9.0)	68 (18.6)	< 0.001
*Resuscitation parameters*					
IHCA, n (%)	183 (27.3)	115 (16.8)	56 (17.4)	59 (16.2)	0.65
No-flow time, min, median (IQR)	2.0 (1.0–5.0)	0 (0–5)	0 (0–0.5)	2 (0–9)	< 0.001
Low-flow time, min, median (IQR)	20 (10–32)	15 (10–25)	10 (5–20)	20 (11–30)	< 0.001
Witnessed cardiac arrest	460 (68.6)	555 (80.9)	292 (91.0)	263 (72.1)	< 0.001
Bystander CPR	433 (64.5)	487 (71.0)	270 (84.1)	217 (59.5)	< 0.001
*Initial rhythm*, n (%)					< 0.001
VF	203 (30.3)	330 (48.2)	216 (67.3)	114 (31.3)	
VT		32 (4.7)	18 (5.6)	14 (3.8)	
PEA	468 (69.7)	154 (22.5)	34 (10.6)	120 (33.0)	
Asystole		112 (16.4)	15 (4.7)	97 (26.6)	
Unknown		57 (8.3)	38 (11.8)	19 (5.2)	
*Adrenaline*					< 0.001
No adrenaline	n. a	245 (37.1)	167 (54.6)	78 (22.0)	
> 0 and < 3 mg	n. a	204 (30.9)	81 (26.5)	123 (34.7)	
3 mg and more	n. a	211 (32.0)	58 (19.0)	153 (43.2)	
*Arrest aetiology*					< 0.001
Cardiac cause	358 (53.4)	428 (62.8)	248 (78.5)	180 (49.2)	
Other/unknown	313 (46.6)	253 (37.2)	68 (21.5)	186 (50.8)	
*Clinical/laboratory parameters at ICU admission*					
Glasgow Coma Scale, median (IQR)	3 (3–7)	3 (3–6)	5 (3–14)	3 (3–3)	< 0.001
Reactive pupillary light reflex	367 (54.7)	514 (83.6)	277 (97.2)	237 (71.8)	< 0.001
OHCA score, median (IQR)	34.57 (20.22–47.18)	21 (6–35)	8 (−3–21)	31 (21–45)	< 0.001
CAHP score, median (IQR)	177.31 (122.38–215.78)	160 (121–194)	125 (98–152)	186.50 (160–218)	< 0.001
C-reactive protein, mg/dl, median (IQR)	0.6 (0.5–1.8)	0.50 (0.17–1.75)	0.34 (0.14–0.92)	0.79 (0.22–2.69)	< 0.001
Creatinine, mg/dl, median (IQR)	1.2 (1.0–1.7)	1.12 (0.88–1.4)	1.04 (0.87–1.22)	1.20 (0.93–1.56)	< 0.001
Glucose, mg/dl, median (IQR)	230 (166–309)	172.8 (133.2–241.2)	153.9 (124.2–199.8)	192.6 (147.6–264.6)	< 0.001
Potassium, mmol/l, median (IQR)	4.1 (3.6–5.1)	4.2 (3.8–4.7)	4.2 (3.8–4.7)	4.3 (3.8–4.8)	0.31
Phosphate, mmol/l, median (IQR)	2.03 (1.42–2.68)	1.38 (1.06–1.97)	1.19 (0.97–1.51)	1.66 (1.25–2.40)	< 0.001
pH, median (IQR)	7.16 (6.99–7.32)	7.25 (7.12–7.33)	7.30 (7.22–7.34)	7.20 (7.07–7.29)	< 0.001
Haemoglobin, g/dl, median (IQR)	12.4 (10.0–14.3)	13.5 (12.0–14.9)	13.8 (12.6–15.0)	13.1 (11.2–14.6)	< 0.001
Sodium, mmol/l, median (IQR)	n. a	138 (136–141)	139 (136–141)	138 (135–141)	0.038
Lactate, mmol/l, median (IQR)	8.7 (5.5–13.1)	5.6 (2.9–9.0)	3.9 (2.1–6.2)	7.5 (4.9–10.3)	< 0.001
*Post-cardiac arrest intervention*, *n (%)*					
Intubation	629 (93.7)	569 (82.8)	226 (70.4)	343 (93.7)	< 0.001
Targeted temperature management	347 (51.7)	351 (51.1)	150 (46.7)	201 (54.9)	0.032
Pharmacological haemodynamic support	n. a	556 (80.9)	238 (74.1)	318 (86.9)	< 0.001
Coronary angiography	276 (41.1)	444 (64.7)	256 (79.8)	188 (51.5)	< 0.001

*CAHP* cardiac arrest hospital prognosis score, *COPD* chronic obstructive pulmonary disease, *CPC* cerebral performance category scale, *CPR* cardiopulmonary resuscitation, *IHCA* In-hospital cardiac arrest, *IQR* inter-quartile range, *n.a.* not available, *OHCA* out-of-hospital cardiac arrest score, *PEA* pulseless electrical activity, *PROLOGUE* prognostication using logistic regression model for unselected adult cardiac arrest patients in the early stages, *PROPHETIC* prognostication of outcome in patients with out-of hospital cardiac arrest hospitalised in intensive care, *SD* standard deviation, *VF* ventricular fibrillation, *VT* ventricular tachycardia

### Neurological outcome and mortality

Three-hundred and twenty-one patients (46.7%) survived to hospital discharge with good neurological outcome, 68 (9.9%) survived with poor neurological outcome and 298 (43.4%) died. A Kaplan–Meier survival estimate of the whole population is shown in Additional file 1: Figure S1.

### Score performance

**Fig. 1:**
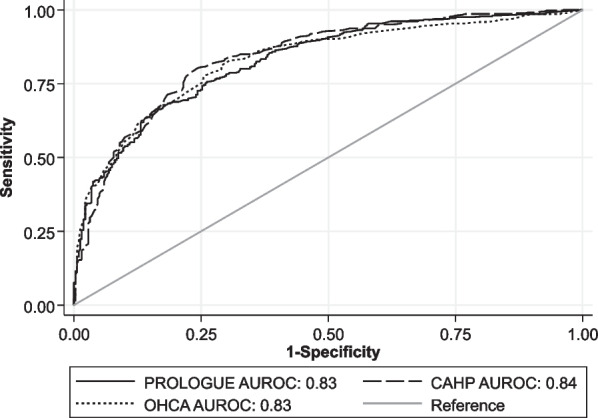
Comparison of ROC curves of the PROLOGUE, OHCA and CAHP scores for the primary endpoint. The differences between the three scores were not statistically significant (*p* = 0.495). AUROC Area under the receiver operating characteristic curve; CAHP Cardiac arrest hospital prognosis; OHCA Out-of-hospital cardiac arrest score; PROLOGUE Prognostication using logistic regression model for unselected adult cardiac arrest patients in the early stages

**Fig. 2:**
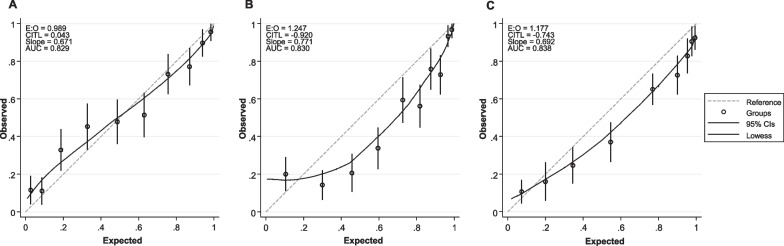
Calibration plots of the PROLOGUE (**A**), OHCA (**B**) and CAHP (**C**) scores for the primary endpoint. AUC Area under the receiver operating characteristic curve; CAHP Cardiac arrest hospital prognosis; CITL Calibration in the large; E:O Expected vs. observed ratio of poor outcome OHCA Out-of-hospital cardiac arrest score; PROLOGUE Prognostication using logistic regression model for unselected adult cardiac arrest patients in the early stages

The prognostic performance of the PROLOGUE, OHCA and CAHP scores for the primary and secondary outcome are summarised in Table 2. The PROLOGUE score showed an AUROC of 0.83 (95% CI 0.80 to 0.86) and good overall calibration for the prediction of poor neurological outcome at hospital discharge. The AUROC of the OHCA and CAHP scores were 0.83 (95% CI 0.80 to 0.86) and 0.84 (95% CI 0.81 to 0.87) respectively. The differences between the AUROC of all three scores were not significant (*p* = 0.495). For the primary endpoint, a graphical comparison of the ROC of the three scores is shown in Fig. 1. The calibration of the OHCA score was poor with overestimation of poor outcome, the CAHP score showed acceptable calibration, also with a tendency to overestimate poor outcome. Calibration plots of all three scores for the primary outcome are shown in Fig. 2. The PROLOGUE score showed the highest AUROC for the prognostication of in-hospital mortality, however, the differences between the three scores were not significant (*p* = 0.275). The ROC curves and calibration plots for the secondary outcome are shown in Additional file 1: Figures S2 and S3 respectively. The highest decile of the PROLOGUE score’s predicted risk (≥ 0.9) predicted poor neurological outcome with a specificity of 99.1%, but poor sensitivity of 17.8%. The prognostic accuracy of the PROLOGUE score at each decile of predicted risk is shown in Table 3. A Kaplan–Meier survival estimate stratified by quartiles of risk of poor outcome as predicted by the PROLOGUE score is shown in Additional file 1: Figure S4. The prognostic accuracy of the OHCA and CAHP scores at the pre-defined cut-offs are shown in Additional file 1: Tables S1 and S2, Kaplan–Meier survival estimates stratified by OHCA and CAHP risk categories in Additional file 1: Figures S5 and S6 respectively. The PROLOGUE score performed similarly in OHCA and IHCA patients (AUROC 0.83 [95% CI 0.80 to 0.87] vs. 0.80 [95% CI 0.72 to 0.88], *p* = 0.437) as well as in men and women (AUROC 0.83 [95% CI 0.80 to 0.87] vs. 0.82 [95% CI 0.76 to 0.88], *p* = 0.777). The OHCA and CAHP scores performed similarly in men and women, but significantly worse in IHCA patients than in OHCA patients (AUROC 0.75 [95% CI 0.66 to 0.84] vs. 0.85 [95% CI 0.82 to 0.88], *p* = 0.045 and 0.76 [95% CI 0.67 to 0.85] vs. 0.86 [95% CI 0.83 to 0.89], *p* = 0.049 respectively). The results of all subgroup analyses are summarised in Additional file 1: Table 3.

**Table 2:** Comparison between scores

Score	OHCA	CAHP	PROLOGUE*
*A: Neurological outcome at hospital discharge*			
Score points in patients with good neurological outcome, n = 321	8 ([−3]–21)	125 (98–152)	0.21 (0.07–0.54)
Score points in patients with poor neurological outcome, n = 366	31 (21–45)	186.5 (160–218)	0.82 (0.52–0.95)
*p*-value (Wilcoxon rank-sum test)	< 0.001	< 0.001	< 0.001
OR per decile increase (95% CI)	1.66 (1.54–1.79)	1.71 (1.58–1.85)	1.65 (1.53–1.78)
AUROC (95% CI)	0.83 (0.80–0.86)	0.84 (0.81–0.87)	0.83 (0.80–0.86)
*B: Mortality at hospital discharge*			
Score points in survivors, n = 390	10 ([−2]–23)	132 (103–163)	0.26 (0.08–0.58)
Score points in non-survivors, n = 297	34 (22–46)	189 (166–222)	0.86 (0.65–0.95)
*p*-value (Wilcoxon rank-sum test)	< 0.001	< 0.001	< 0.001
OR per decile increase (95% CI)	1.64 (1.52–1.76)	1.67 (1.55–1.81)	1.74 (1.60–1.89)
AUROC (95% CI)	0.82 (0.79–0.85)	0.83 (0.80–0.86)	0.85 (0.82–0.88)

*For the PROLOGUE score the predicted probability of poor outcome is provided instead of the score points. Data presented as median (IQR) unless otherwise specified.

*AUROC* area under the receiver operating characteristic curve, *CAHP* cardiac arrest hospital prognosis score, *CI* confidence interval, *OHCA* out-of-hospital cardiac arrest score, *OR* odds ratio, *PROLOGUE* prognostication using logistic regression model for unselected adult cardiac arrest patients in the early stages

**Table 3:** Performance of the PROLOGUE score at different cut-off points

Cut-off (predicted probability of poor outcome)	≥ 0.1	≥ 0.2	≥ 0.3	≥ 0.4	≥ 0.5	≥ 0.6	≥ 0.7	≥ 0.8	≥ 0.9
*A: Neurological outcome at hospital discharge*									
Total number of patients, n	618	546	476	412	343	273	206	136	68
CPC 1–2, n (%)	260 (42.1)	196 (35.9)	149 (31.3)	114 (27.7)	78 (22.7)	44 (16.1)	26 (12.6)	10 (7.4)	3 (4.4)
CPC 3–5, n (%)	358 (57.9)	350 (64.1)	327 (68.7)	298 (72.3)	265 (77.3)	229 (83.9)	180 (87.4)	126 (92.6)	65 (95.6)
Sensitivity, % (95% CI)	97.8 (95.7–99.1)	95.6 (93.0–97.5)	89.3 (85.7–92.3)	81.4 (77.1–85.3)	72.4 (67.5–76.9)	62.6 (57.4–67.5)	49.2 (43.9–54.4)	34.4 (29.6–39.5)	17.8 (14.0–22.1)
Specificity, % (95% CI)	19.0 (14.9–23.7)	38.9 (33.6–44.5)	53.6 (48.0–59.1)	64.5 (59.0–69.7)	75.7 (70.6–80.3)	86.3 (82.0–89.9)	91.9 (88.4–94.6)	96.9 (94.3–98.5)	99.1 (97.3–99.8)
PPV, % (95% CI)	57.9 (53.9–61.9)	64.1 (59.9–68.1)	68.7 (64.3–72.8)	72.3 (67.7–76.6)	77.3 (72.5–81.6)	83.9 (79.0–88.0)	87.4 (82.1–91.6)	92.6 (86.9–96.4)	95.6 (87.6–99.1)
NPV, % (95% CI)	88.4 (78.4–94.9)	88.7 (82.2–93.4)	81.5 (75.6–86.5)	75.3 (69.7–80.3)	70.6 (65.5–75.4)	66.9 (62.1–71.4)	61.3 (56.8–65.7)	56.4 (52.2–60.6)	51.4 (47.4–55.4)

Cut-off (predicted probability of death)	≥ 0.1	≥ 0.2	≥ 0.3	≥ 0.4	≥ 0.5	≥ 0.6	≥ 0.7	≥ 0.8	≥ 0.9
*B: Mortality at hospital discharge*									
Total number of patients, n	618	546	476	412	343	273	206	136	68
Survivors, n (%)	324 (52.4)	258 (47.3)	199 (41.8)	152 (36.9)	105 (30.6)	65 (23.8)	40 (19.4)	18 (13.2)	5 (7.4)
Non-survivors, n (%)	294 (47.6)	288 (52.7)	277 (58.2)	260 (63.1)	238 (69.4)	208 (76.2)	166 (80.6)	118 (86.8)	63 (92.6)
Sensitivity, % (95% CI)	99.0 (97.1–99.8)	97.0 (94.3–98.6)	93.3 (89.8–95.8)	87.5 (83.2–91.1)	80.1 (75.1–84.5)	70.0 (64.5–75.2)	55.9 (50.0–61.6)	39.7 (34.1–45.5)	21.2 (16.7–26.3)
Specificity, % (95% CI)	16.9 (13.3–21.0)	33.8 (29.2–38.8)	49.0 (43.9–54.1)	61.0 (56.0–65.9)	73.1 (68.4–77.4)	83.3 (79.3–86.9)	89.7 (86.3–92.6)	95.4 (92.8–97.2)	98.7 (97.0–99.6)
PPV, % (95% CI)	47.6 (43.6–51.6)	52.7 (48.5–57.0)	58.2 (53.6–62.7)	63.1 (58.2–67.8)	69.4 (64.2–74.2)	76.2 (70.7–81.1)	80.6 (74.5–85.8)	86.8 (79.9–92.0)	92.6 (83.7–97.6)
NPV, % (95% CI)	95.7 (87.8–99.1)	93.6 (88.2–97.0)	90.5 (85.7–94.1)	86.5 (81.9–90.3)	82.8 (78.4–86.7)	78.5 (74.2–82.4)	72.8 (68.6–76.7)	67.5 (63.4–71.4)	62.2 (58.2–66.0)

*CI* confidence interval, *CPC* cerebral performance category scale, *NPV* negative predictive value, *PPV* positive predictive value

## Discussion

This study aimed to externally validate the PROLOGUE score in a large, unselected population of cardiac arrest patients and to compare it to the two thoroughly validated scoring systems OHCA and CAHP for the prognostication of neurological outcome after cardiac arrest. All scores showed good prognostic accuracy in our cohort, with the differences between the score’s performances being minor and not statistically significant. In our sample, the PROLOGUE score was well-calibrated. The OHCA and CAHP score in contrast both showed a tendency to overestimate poor outcome. This is a major limitation of the OHCA and CAHP scores, as overestimation of poor outcomes might lead to premature withdrawal of life-sustaining treatment in patients who otherwise might have survived with a good neurological outcome.

To our knowledge, this study is the third external validation of the PROLOGUE score overall and the second one performed in Europe [30, 31]. Our findings are in line with the other European validation study performed in Austria, in which the score showed an AUROC of 0.82 (95% CI 0.80 to 0.85) [31]. In both European studies, the PROLOGUE score failed to reach the outstanding performance it showed in the internal validation and one external validation, both conducted in South Korea. There are some possible explanations for the difference in prognostic accuracy between the South Korean and the European studies. First, there are important differences in the baseline characteristics and predictor values between the development cohort and our study sample including higher proportions of IHCA patients and lower proportions of shockable initial rhythm, witnessed cardiac arrests and cardiac aetiology in the South Korean studies. Second, the proportion of poor neurological outcome at hospital discharge was higher in the South Korean studies (64.3 to 69.3% in the South Korean cohorts vs. 53.3% in our cohort) which might be due to said differences between the populations studied and/or differences in the clinical management of the patients. Third, none of the patients in the South Korean studies underwent withdrawal of life-sustaining therapy (WLST), since this was not allowed in South Korea until 2018 [14]. Fourth, pre-hospital management of cardiac arrest and the organisation of the rescue chains might differ substantially between countries. All of this highlights the importance of external validation of scoring systems such as the PROLOGUE, OHCA and CAHP scores before applying them in a region or country different from where it was developed. Hence, before applying the PROLOGUE score in Europe, North America or South America further external validation studies will be necessary.

However, the PROLOGUE score has some advantages over the OHCA and CAHP scores: First, it does not include the no-flow time, which is often missing or incorrectly estimated in clinical routine because cardiac arrests are often not witnessed. Second, it was explicitly developed for use in IHCA and OHCA patients. Thus, it can be applied to unselected cardiac arrest patients, whereas the OHCA and CAHP score were developed for OHCA patients only and consequentially showed significantly worse performance when applied to IHCA patients in the subgroup analysis—a finding which is in line with previous publications [16, 39]. The PROLOGUE score thus might be a promising alternative to the OHCA and CAHP scores mainly due to its better clinical applicability by omitting the no-flow time as a parameter and broader suitability to OHCA and IHCA survivors with—in our study—similar prognostic accuracy. Further validations are needed to confirm the reliability and generalisability of these findings. As a matter of fact, prognostic scores are not omniscient and should be seen as a decision aid only. In a next step, interventional studies should evaluate if the application of such scores improves patient management [40].

Our study has some limitations. First, a certain risk of self-fulfilling prophecy has to be acknowledged, a limitation that has to be kept in mind always when conducting or interpreting prognostic factor studies [41, 42]. However, this problem is difficult to address since treating physicians cannot be blinded to predictor variables that are a necessary part of clinical decision making such as findings of clinical examination or laboratory values. In our study, the treating physicians had access to all parameters included in the PROLOGUE score individually but not to the score value or the resulting prediction of the probability of poor outcome for their patient. However, clinicians should be aware that clinical prediction models may be statistically valid on average, but for any individual patient, it remains a complex clinical decision based on different parameters. Second, our cohort is from a single centre, thus limiting generalisability to other regions or countries. Third, there were some differences in data acquisition between the original study and ours. Whereas in the Korean development cohort the first available values after hospital admission were used for the calculation of the score, in our cohort the values nearest to ICU admission were used. Thus, in our study, the parameters might tend to have been assessed a little later, which might be the reason for some differences in predictor values between our population and the South Korean development cohort. However, such differences can always occur when a scoring system is applied in a different setting or hospital, which is why we recommend validating and—if necessary—recalibrating the score before application in a certain population and setting. Fourth, in Switzerland if poor outcome is evident early in the treatment course (e.g., brain herniation due to excessive cerebral edema) life-supporting measures are frequently withdrawn and changed to a palliative regimen. This might explain the rather low number of poor neurological outcomes (n = 68, 9.9%) in our cohort. In general, one should aim for ≥ 100 events of a particular outcome for validation, which might reduce the certainty of our results. Fifth, we did not test the results for subgroups based on important socioeconomic factors such as being part of an ethnical minority group or social status. Also, we did not change the model to improve fit, but just validated the original score.

Finally, we focused on poor outcome at hospital discharge as our primary outcome which is in line with the original paper, but did not report other patient-centered outcomes.

Strengths of our study include the large population of unselected cardiac arrest patients and the treatment modalities which are in line with current European guidelines, both of which indicate a high external validity of our results. Furthermore, analysis and reporting of our study followed current methodological guidelines [33], which is essential for the comparability of our result to other studies and for the usability of our results for evidence synthesis in the form of a systematic review and/or meta-analysis of score performance in the future.

## Conclusion

In our prospective cohort of unselected adult cardiac arrest patients, the PROLOGUE, OHCA and CAHP score all showed good prognostic accuracy. The PROLOGUE score performs well in predicting poor neurological outcome in IHCA and OHCA patients and, if validated in qualitative or interventional studies, might support early discussions about goals of care and the extent of therapeutic effort between physicians and next of kin on the ICU. However, the outstanding performance of the PROLOGUE score in the South Korean studies could not be reproduced, which highlights the importance of external validation studies in the evaluation of prognostic models.

## Supplementary Information

**Additional file 1**. Supplementary tables and figures.

## Data Availability

The datasets generated and/or analysed during the current study are available from the corresponding author on reasonable request.

## Abbreviations

APACHE II: Acute physiology and chronic health evaluation II
ANOVA: Analysis of variance
AUROC: Area under the receiver operating characteristic curve
CAHP: Cardiac arrest hospital prognosis
CI: Confidence interval
COMMUNICATE: Communication in out-of hospital cardiac arrest events
COPD: Chronic obstructive pulmonary disease
CPC: Cerebral performance category
CPR: Cardiopulmonary resuscitation
EKNZ: Ethikkommission Nordwest- und Zentralschweiz (Ethics committee of north-western and central Switzerland)
GCS: Glasgow coma scale
ICU: Intensive care unit
IHCA: In-hospital cardiac arrest
IQR: Interquartile range
NPV: Negative predictive value
OHCA: Out-of-hospital cardiac arrest
PPV: Positive predictive value
PROLOGUE: Prognostication using logistic regression model for unselected adult cardiac arrest patients in the early stages
PROPHETIC: Prognostication of outcome in patients with out-of hospital cardiac arrest hospitalized in intensive care
ROC: Receiver operating characteristic curve
ROSC: Return of spontaneous circulation
SAPS II: Simplified acute physiology score II
TRIPOD: Transparent reporting of a multivariable prediction model for individual prognosis or diagnosis
TTM: Targeted temperature management
