# Supplementary material for: External validation of the PROLOGUE score to predict neurological outcome in adult patients after cardiac arrest: a prospective cohort study
Source: Scand J Trauma Resusc Emerg Med. 2023 Apr 4;31:16. doi: 10.1186/s13049-023-01081-1 (PMC10074653; doi:10.1186/s13049-023-01081-1)
Supplement: Supplementary file 1 — Additional file 1. Supplementary tables and figures. [file 13049_2023_1081_MOESM1_ESM.pdf]

Supplementary Table 1. Performance of OHCA score at pre-defined cut-offs

**A: Neurological outcome at hospital discharge**			
**OHCA Category**	**>I**	**>II**	**>III**
	cut-off 20 points	cut-off 40 points	cut-off 60 points
Total number of patients, n	357	136	11
CPC 1-2, n (%)	81 (22.7)	7 (5.1)	1 (9.1)
CPC 3-5, n (%)	276 (77.3)	129 (94.9)	10 (90.9)
Sensitivity, % (95 % CI)	75.4 (70.7-79.7)	35.2 (30.4-40.4)	2.7 (1.3-5.0)
Specificity, % (95 % CI)	74.8 (69.6-79.4)	97.8 (95.6-99.1)	99.7 (98.3-100.0)
PPV, % (95 % CI)	77.3 (72.6-81.6)	94.9 (89.7-97.9)	90.9 (58.7-99.8)
NPV, % (95 % CI)	72.7 (67.6-77.5)	57.0 (52.7-61.2)	47.3 (43.5-51.2)
**B: Mortality at hospital discharge**			
**OHCA Category**	**>I**	**>II**	**>III**
	cut-off 20 points	cut-off 40 points	cut-off 60 points
Total number of patients, n	357	136	11
Survivors, n (%)	124 (34.7)	15 (11.0)	2 (18.2)
Non-survivors, n (%)	233 (65.3)	121 (89.0)	9 (81.8)
Sensitivity, % (95 % CI)	78.5 (73.3-83.0)	40.7 (35.1-46.6)	3.0 (1.4-5.7)
Specificity, % (95 % CI)	68.2 (63.3-72.8)	96.2 (93.7-97.8)	99.5 (98.2-99.9)
PPV, % (95 % CI)	65.3 (60.1-70.2)	89.0 (82.5-93.7)	81.8 (48.2-97.7)
NPV, % (95 % CI)	80.6 (75.9-84.7)	68.1 (64.0-71.9)	57.4 (53.6-61.2)

CI Confidence interval; **CPC** Cerebral performance category scale; **NPV** Negative predictive value; **OHCA** Out-of hospital cardiac arrest score; **PPV** Positive predictive value

Supplementary Table 2. Performance of CAHP score at pre-defined cut-offs		
**A: Neurological outcome at hospital discharge**		
**CAHP Category**	**>I**	**>II**
	cut-off 150 points	cut-off 200 points
Total number of patients, n	381	147
CPC 1-2, n (%)	84 (22.1)	15 (10.2)
CPC 3-5, n (%)	297 (77.9)	132 (89.8)
Sensitivity, % (95 % CI)	81.1 (76.8-85.0)	36.1 (31.1-41.2)
Specificity, % (95 % CI)	73.8 (68.7-78.6)	95.3 (92.4-97.4)
PPV, % (95 % CI)	78.0 (73.4-82.0)	89.8 (83.7-94.2)
NPV, % (95 % CI)	77.5 (72.4-82.0)	56.7 (52.4-60.9)
**B: Mortality at hospital discharge**		
**CAHP Category**	**>I**	**>II**
	cut-off 150 points	cut-off 200 points
Total number of patients, n	381	147
Survivors, n (%)	131 (34.4)	28 (19.1)
Non-survivors, n (%)	250 (65.6)	119 (80.9)
Sensitivity, % (95 % CI)	84.2 (79.5-88.1)	40.1 (34.4-45.9)
Specificity, % (95 % CI)	66.4 (61.5-71.1)	92.8 (89.8-95.2)
PPV, % (95 % CI)	65.6 (60.6-70.4)	81.0 (73.7-87.0)
NPV, % (95 % CI)	84.6 (80.1-88.5)	67.0 (62.9-71.0)
**CAHP** Cardiac arrest hospital prognosis score; **CI** Confidence interval; **CPC** Cerebral performance category scale; **NPV** Negative predictive value; **PPV** Positive predictive value		

**Supplementary Table 3. Subgroup analyses**			
**Score**	**Subgroup**		**p-value**
	OHCA	IHCA	
OHCA, AUROC (95 % CI)	0.85 (0.82-0.88)	0.75 (0.66-0.84)	0.045
CAHP, AUROC (95 % CI)	0.86 (0.83-0.89)	0.76 (0.67-0.85)	0.049
PROLOGUE, AUROC (95 % CI)	0.83 (0.80-0.87)	0.80 (0.72-0.88)	0.437
	Male	Female	
OHCA, AUROC (95 % CI)	0.84 (0.80-0.88)	0.81 (0.75-0.87)	0.430
CAHP, AUROC (95 % CI)	0.84 (0.81-0.88)	0.82 (0.76-0.88)	0.547
PROLOGUE, AUROC (95 % CI)	0.83 (0.80-0.87)	0.82 (0.76-0.88)	0.777
Score performance in the subgroups of OHCA patients vs. IHCA patients and male vs. female gender. Statistical comparison between AUROC was conducted according to DeLong et al. (1988). **AUROC** Area under the receiver operating characteristic curve; **CAHP** Cardiac arrest hospital prognosis score; **CI** Confidence interval; **IHCA** In-hospital cardiac arrest; **OHCA** Out-of hospital cardiac arrest score; **PROLOGUE** Prognostication using logistic regression model for unselected adult cardiac arrest patients in the early stages			

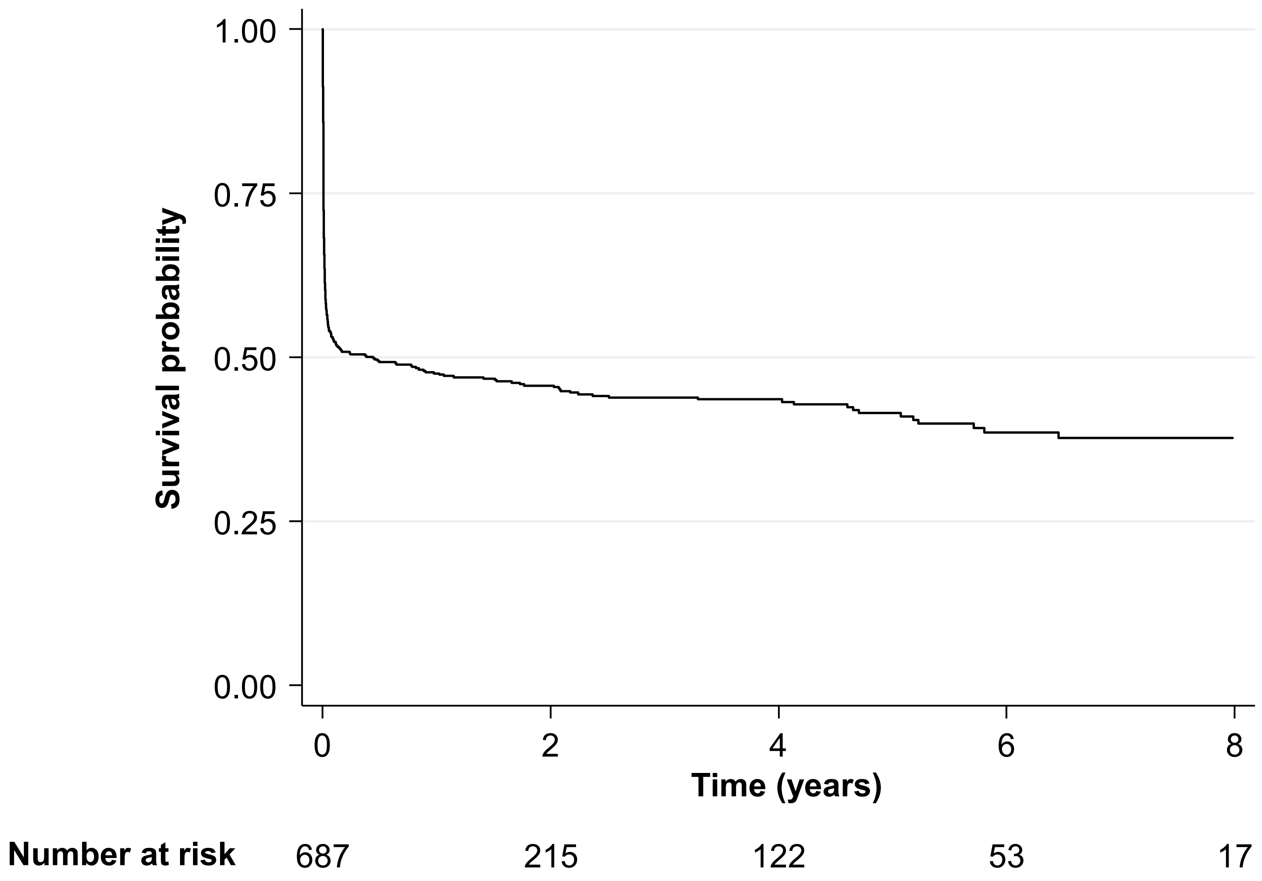

Suppl. Figure 1. Kaplan-Meier survival estimate of the entire cohort with numbers at risk for each time point.

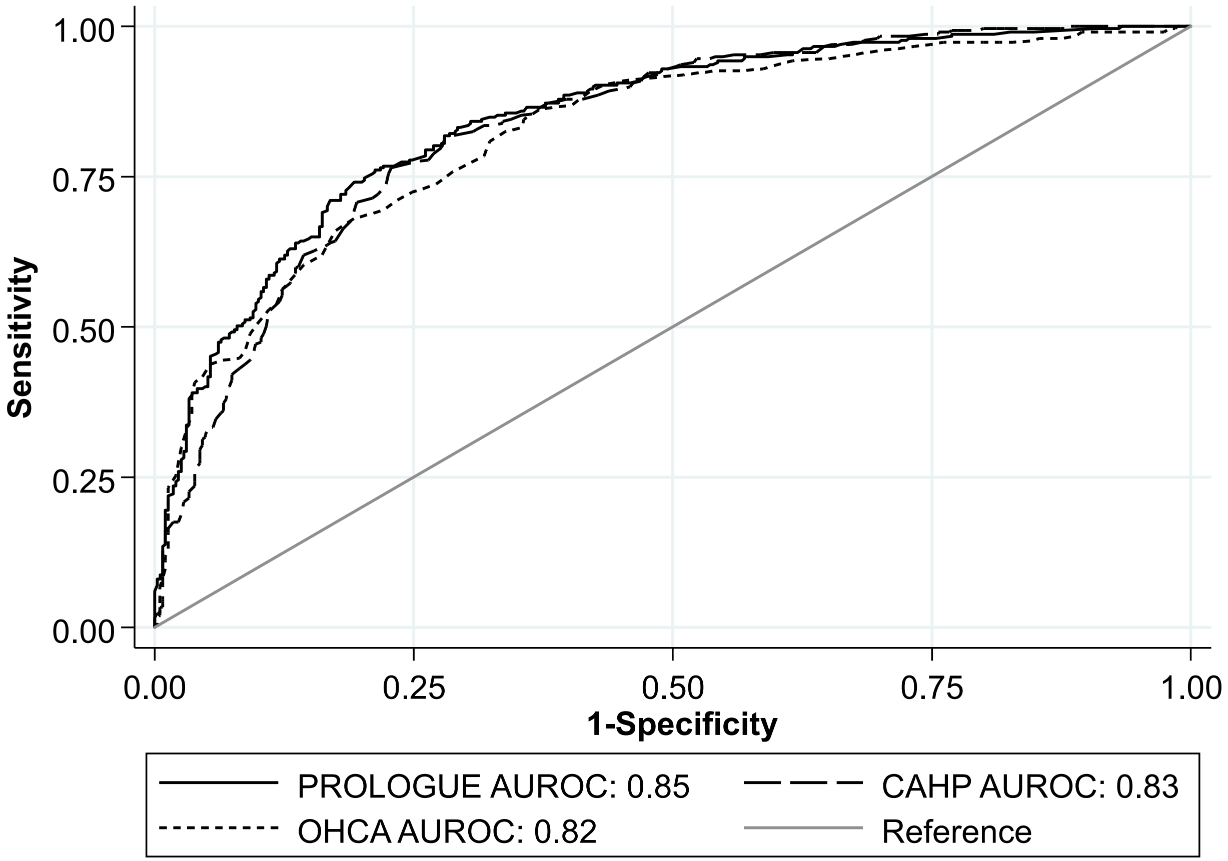

Suppl. Figure 2. Comparison of ROC curves of the PROLOGUE, OHCA and CAHP scores for the secondary endpoint. **AUROC** Area under the receiver operating characteristic curve; **CAHP** Cardiac arrest hospital prognosis; **OHCA** Out-of-hospital cardiac arrest score; **PROLOGUE** Prognostication using logistic regression model for unselected adult cardiac arrest patients in the early stages.

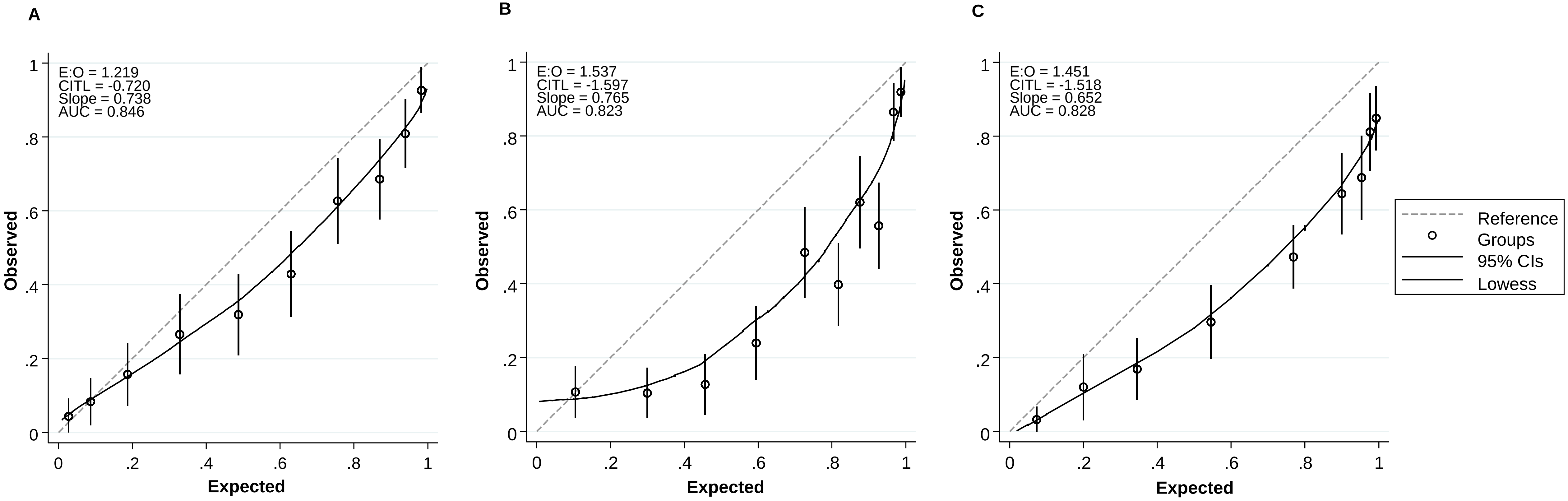

Suppl. Figure 3. Calibration plots of the PROLOGUE (A), OHCA (B) and CAHP (C) scores for the prediction of in-hospital mortality. **AUC** Area under the receiver operating characteristic curve; **CAHP** Cardiac arrest hospital prognosis; **CITL** Calibration in the large; **E:O** Expected vs. observed ratio of in-hospital mortality; **OHCA** Out-of-hospital cardiac arrest score; **PROLOGUE** Prognostication using logistic regression model for unselected adult cardiac arrest patients in the early stages.

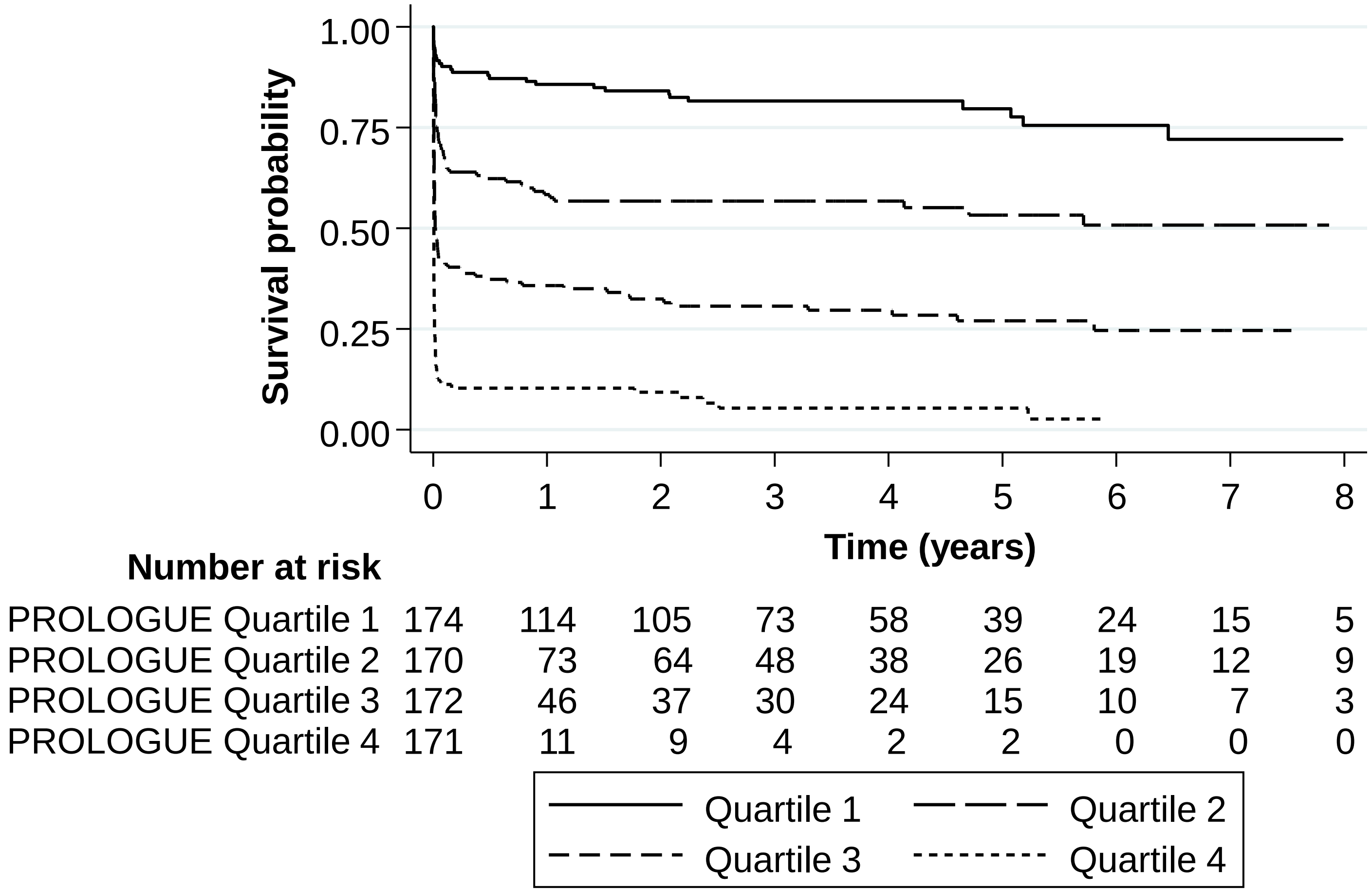

Suppl. Figure 4. Kaplan-Meier survival estimate stratified by quartiles of risk of poor outcome as predicted by the PROLOGUE score.

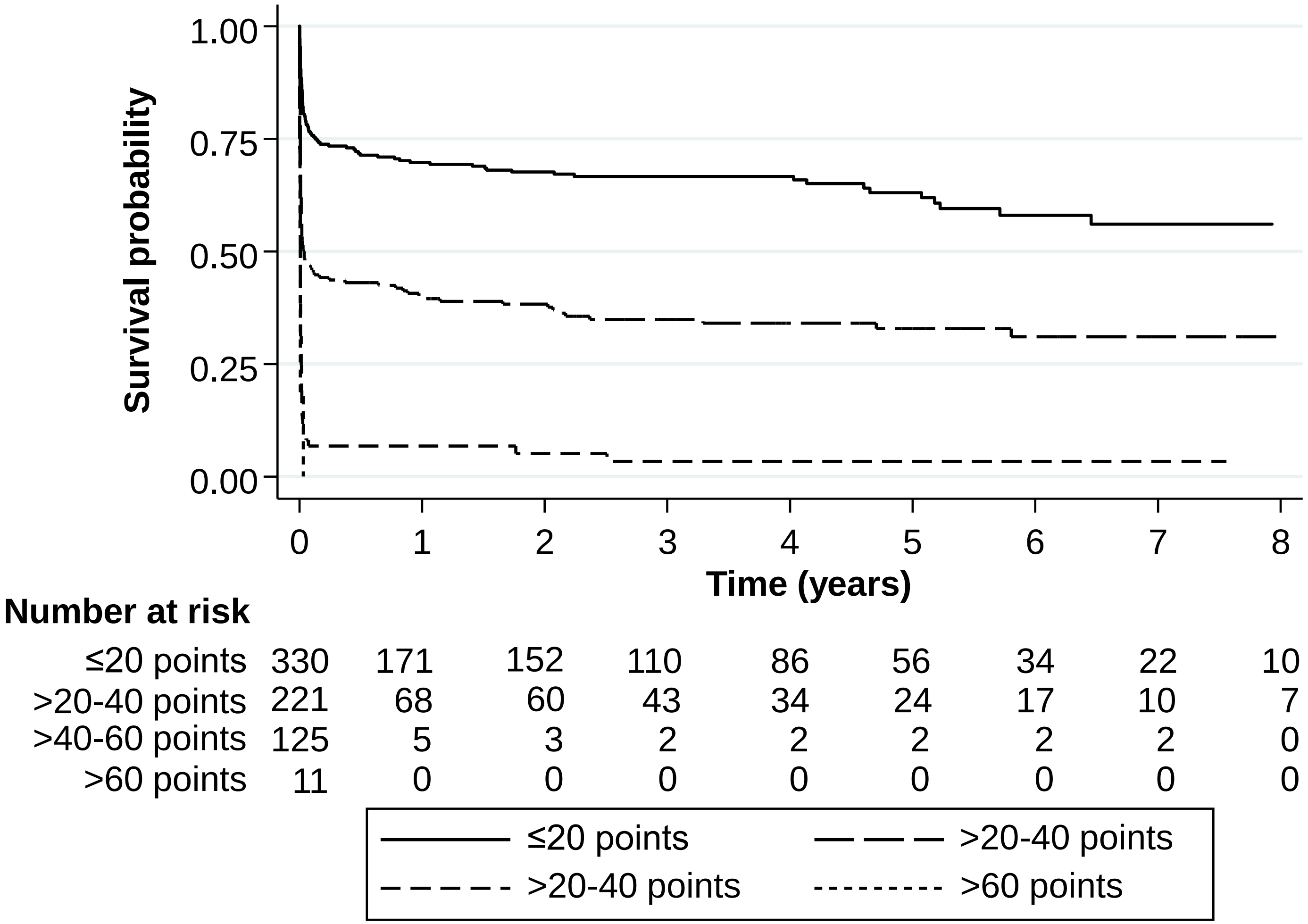

Suppl. Figure 5. Kaplan-Meier survival estimate stratified by OHCA score risk category.

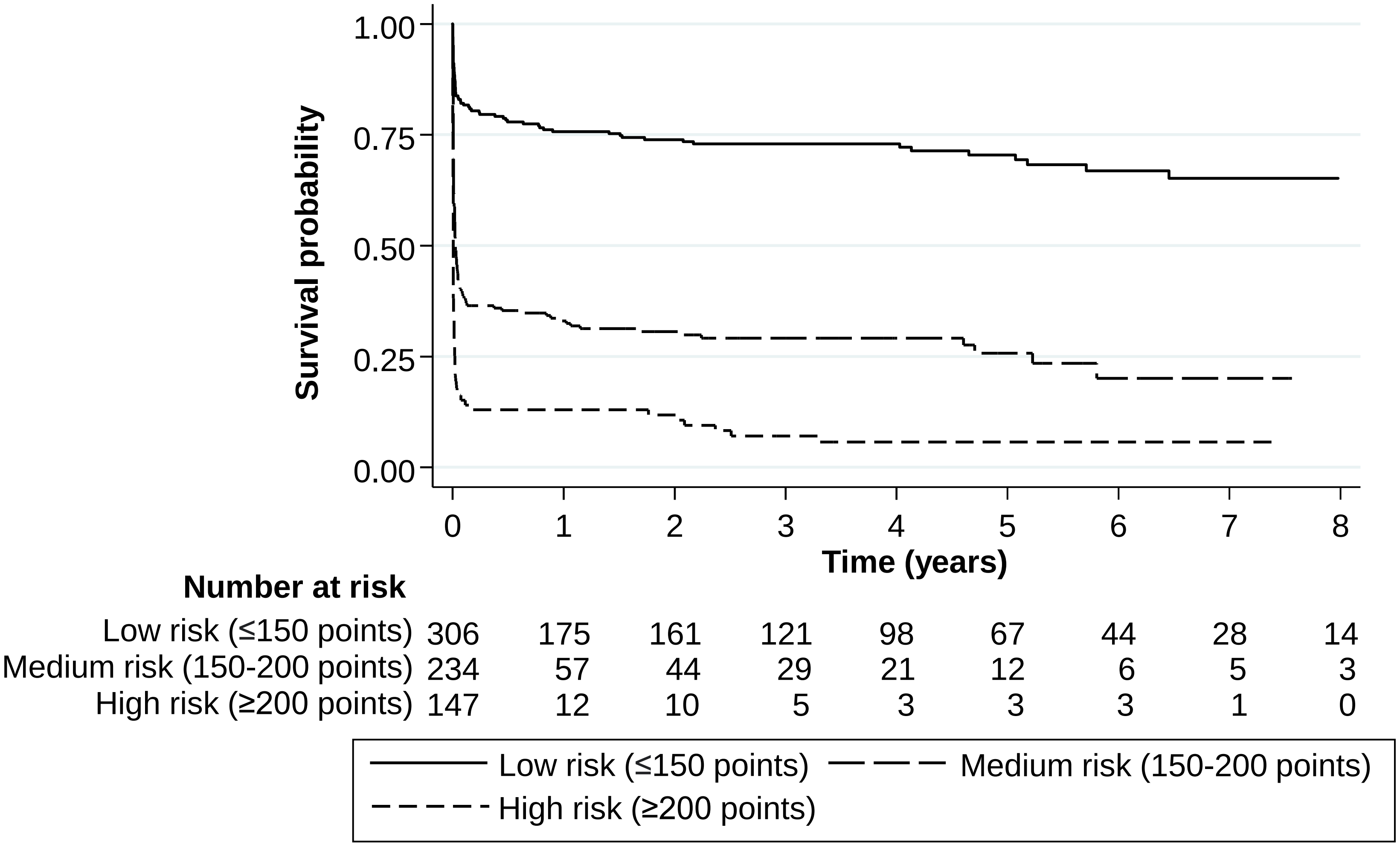

Suppl. Figure 6. Kaplan-Meier survival estimate stratified by CAHP score risk category.
